# Supplementary material for: Label-Free Liquid Chromatography–Mass Spectrometry Quantitation of Relative N- and O-Glycan Concentrations in Human Milk in Japan
Source: Int J Mol Sci. 2024 Feb 1;25(3):1772. doi: 10.3390/ijms25031772 (PMC10855831; doi:10.3390/ijms25031772)
Supplement: Supplementary file 1 [file ijms-25-01772-s001.zip › ijms-2822116-supplementary.pdf]

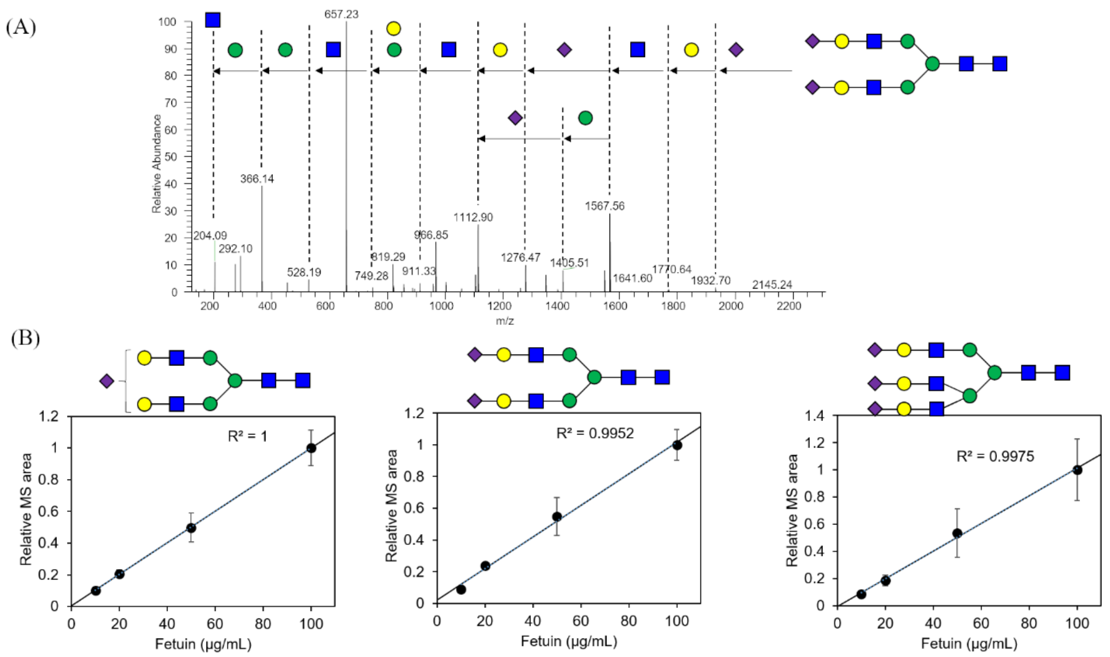

Figure S1. Label-free LC/MS analysis of bovine fetuin *N*-glycan. (A) MS/MS spectrum disialylated biantennary complex-type glycan. (B) Relationship between LC/MS area of three representative *N*-glycans of fetuin and the concentration of fetuin. The relative LC/MS area was set to 1 when 100 μg/mL of fetuin was used. The color symbols are as follows: green circle, Man; yellow square, GalNAc; yellow circle, Gal; blue square, GlcNAc, purple diamond, Neu5Ac.

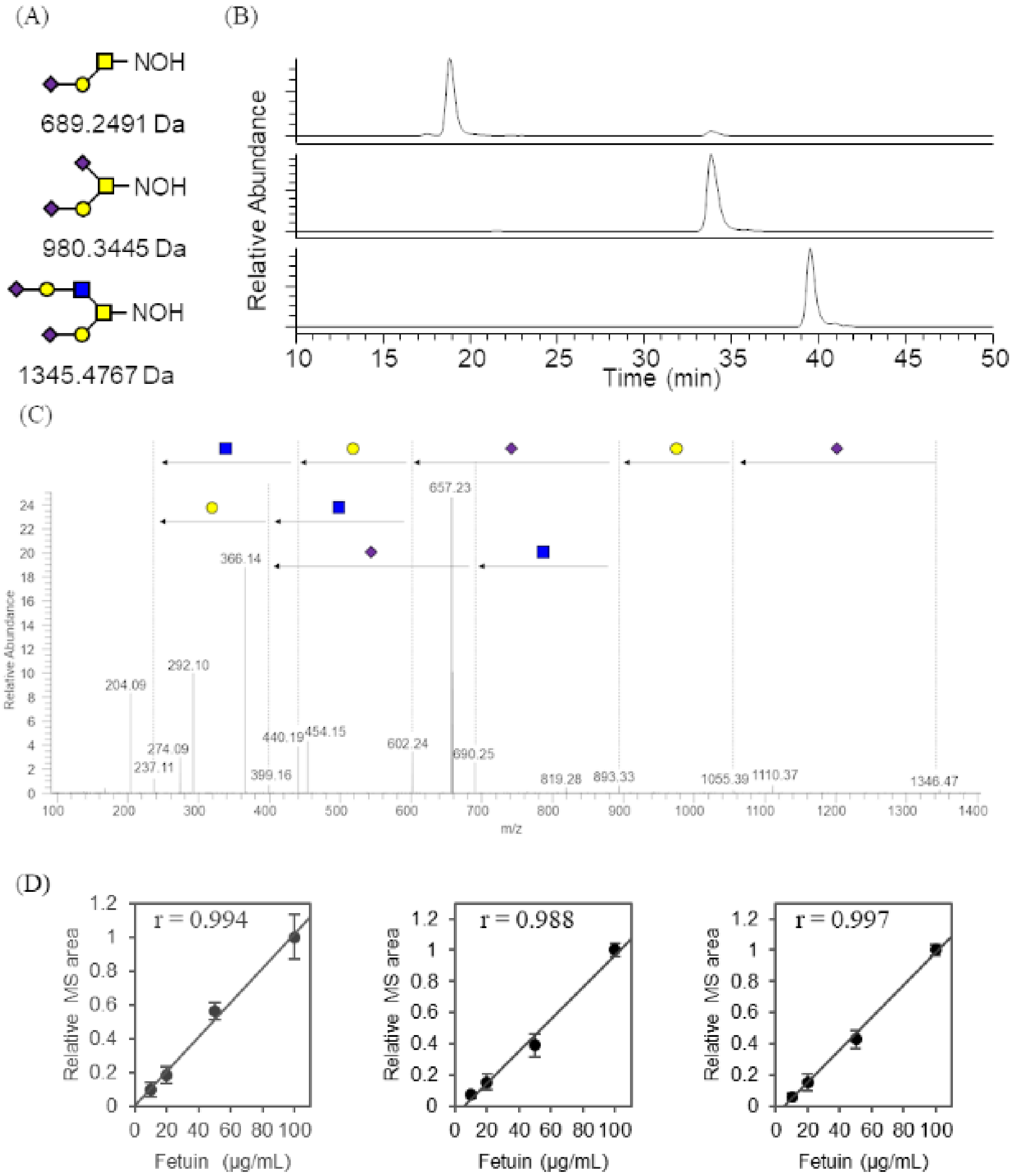

Figure S2. Label-free LC/MS analysis of bovine fetuin *O*-glycan. (A) Structures of oximated *O*-glycans released from bovine fetuin by eliminative oximation. The reducing end of released *O*-glycan is oximated (NOH). The numbers below the structures indicate the monoisotopic mass of each *O*-glycan. (B) Extracted ion current chromatograms at *m/z* 690.25 (top), 981.35 (middle), and 1346.48 (bottom). Oximated *O*-glycans released from 100 μg/mL of fetuin solution were applied to GlycanPac AXH-1 (Thermo Fisher Scientific) and eluted with ammonium formate (pH 4.4)/acetonitrile at a flow rate of 0.4 mL/min. MS chromatogram was acquired in the range of *m/z* 400-2000. (C) MS/MS spectrum at *m/z* 1346.48 corresponding to

Neu5Acα2,3Galβ1,4(Neu5Acα2,3Galβ1,4GlcNAcβ1,6)GalNAc. (D) Relationship between LC/MS area of oximated *O*-glycans with *m/z* 690.25 (left), 981.35 (center), and 1346.48 (right) and the fetuin concentration. The relative LC/MS area was set to 1 when 100 μg/mL of fetuin was used. The color symbols are as follows: yellow square, GalNAc; yellow circle, Gal; blue square, GlcNAc, purple diamond, Neu5Ac.

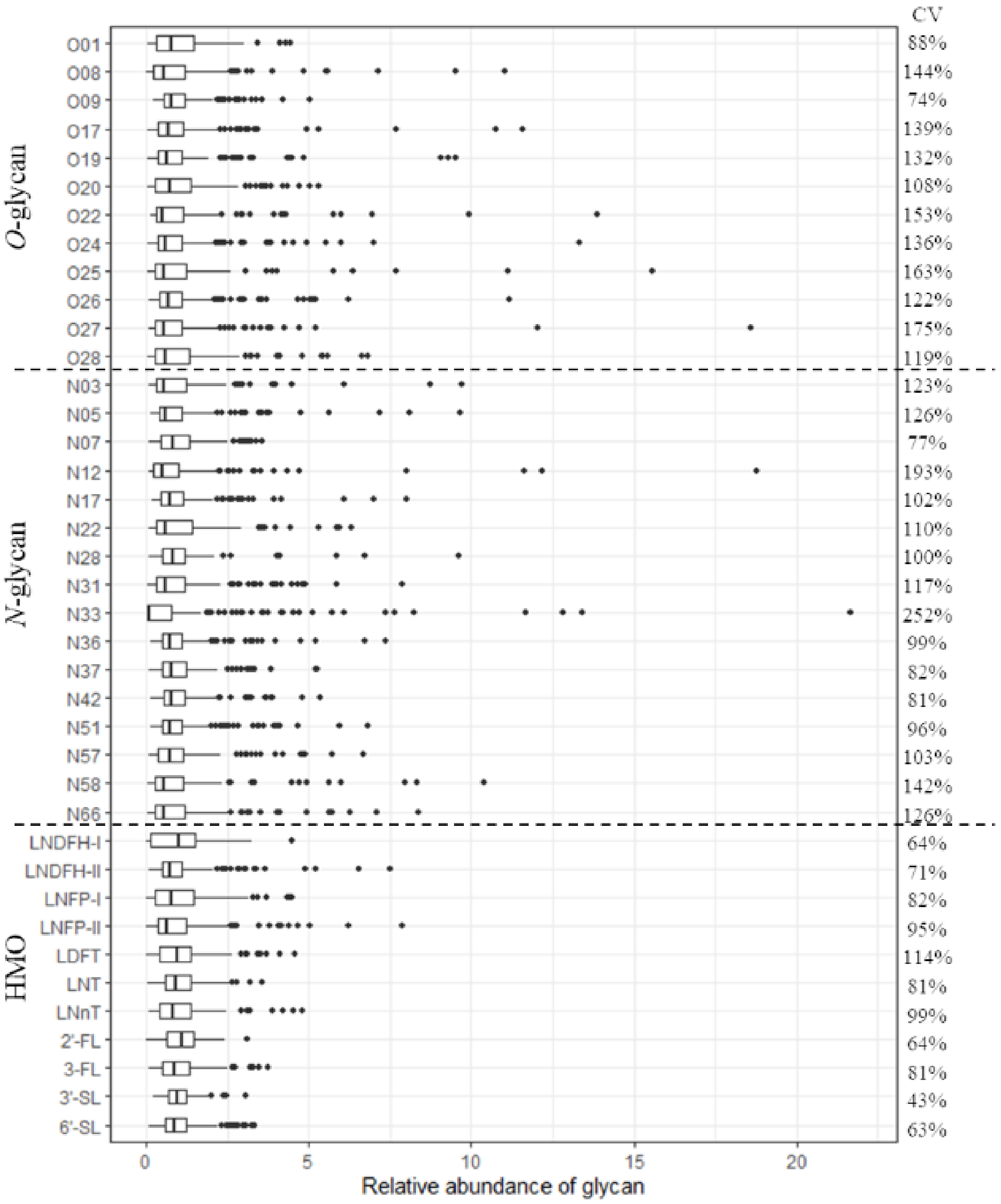

Figure S3. Relative concentration of *N*-glycans, *O*-glycans, and HMOs in 200 samples of human milk. The relative concentration of each glycan was normalized to a mean of 1. The numbers on the right side of the box plot represent the coefficient of variation (CV) of each glycan

Table S1. Concentration of *N*-glycans and *O*-glycans*, and absolute concentration of HMOs (mg/L) in 200 samples of human milk

	Mean	Range	SD	Median	IQR**	skewness
O-glycan						
O01	3.12	0.087-13.9	2.76	2.314	0.906-4.515	1.27
O08	0.465	0.002-5.13	0.67	0.245	0.097-0.537	3.79
O09	39.3	7.51-197.9	29.2	29.637	21.631-46.769	2.39
O17	2.15	0.063-25.0	2.99	1.412	0.777-2.403	4.95
O19	30.5	0.706-290.1	40.1	18.463	11.196-32.375	4.30
O20	1.48	0.019-7.83	1.59	1.04	0.386-1.973	1.73
O22	5.39	0.531-74.7	8.25	2.602	1.619-5.996	4.87
O24	15.3	0.211-203.7	20.9	8.845	5.635-16.404	5.03
O25	55.3	0.873-860.6	89.9	29.888	13.475-68.679	5.62
O26	3.12	0.209-34.8	3.80	2.104	1.282-3.377	4.41
O27	0.312	0.017-5.81	0.545	0.169	0.083-0.334	6.75
O28	0.689	0.007-4.69	0.819	0.399	0.18-0.899	2.44
N-glycan						
N03	0.194	0.018-1.89	0.239	0.108	0.063-0.238	3.86
N05	0.034	0.004-0.328	0.043	0.020	0.013-0.037	3.89
N07	0.032	0.002-0.115	0.025	0.027	0.014-0.042	1.35
N12	0.424	0.023-7.96	0.817	0.213	0.095-0.428	6.16
N17	0.070	0.013-0.558	0.071	0.050	0.03-0.078	3.77
N22	0.166	0.012-1.05	0.182	0.095	0.05-0.23	2.41
N28	0.495	0.032-4.76	0.496	0.395	0.247-0.591	5.05
N31	0.440	0.010-3.46	0.514	0.258	0.13-0.519	2.57
N33	1.16	0.013-25.2	2.94	0.085	0.046-0.881	4.75
N36	0.983	0.096-7.24	0.969	0.691	0.477-1.066	3.53
N37	0.570	0.048-2.99	0.469	0.422	0.28-0.704	2.29
N42	3.43	0.407-18.3	2.76	2.65	1.815-4.082	2.58
N51	0.612	0.072-4.18	0.585	0.432	0.3-0.657	3.03
N57	1.10	0.100-7.33	1.13	0.765	0.403-1.255	2.70
N58	0.184	0.004-1.92	0.262	0.103	0.047-0.206	3.69
N66	0.239	0.006-2.00	0.301	0.133	0.066-0.285	2.94
HMO						
LNDFH-I	299.6	0-1335.5	244.0	296.6	32.8-448	0.72
LNDFH-II	119.3	8.4-893.2	118.1	85.0	55.9-130.5	3.47

LNFP-I	331.5	0.70-1485.8	315.5	254.5	85.5-482	1.32
LNFP-II	522.9	0-4123	593.8	332.4	172-642.9	2.62
LDFT	186.6	0-855.5	153.3	178.3	73.4-252.3	1.33
LNT	619.5	17.4-2211	398.6	558.4	345.2-841.8	1.02
LNnT	73.7	4.7-354.1	60.0	58.1	30.1-101.4	1.82
2'-FL	1419	0-4426.7	912.1	1517.3	898.3-2053.3	-0.03
3-FL	625.3	38.4-2341.3	444.9	520.1	303.8-820.2	1.38
3'-SL	67.4	14.5-206.1	28.9	63.9	46-81.1	1.22
6'-SL	113.2	9.8-376.9	70.8	96.6	65.5-140.6	1.51

*The concentration of *N*-glycan and *O*-glycan was LC-MS area corrected by that of an internal standard.

** IQR, interquartile range

Table S2. Normalized concentration of *N*-glycans, *O*-glycans, and HMOs in 200 samples of human milk*

	range	SD	median	IQR**	skewness
O-glycan					
O01	0.028–4.44	0.884	0.741	0.290–1.446	1.27
O08	0.005–11.0	1.442	0.527	0.208–1.154	3.79
O09	0.191–5.03	0.741	0.754	0.550–1.189	2.39
O17	0.029–11.6	1.390	0.656	0.361–1.117	4.95
O19	0.023–9.53	1.315	0.606	0.368–1.063	4.30
O20	0.013–5.31	1.080	0.704	0.262–1.337	1.73
O22	0.098–13.9	1.531	0.483	0.300–1.113	4.87
O24	0.014–13.3	1.364	0.578	0.368–1.072	5.03
O25	0.016–15.6	1.626	0.540	0.244–1.242	5.62
O26	0.067–11.1	1.217	0.674	0.411–1.082	4.41
O27	0.056–18.6	1.747	0.541	0.266–1.073	6.75
O28	0.011–6.81	1.190	0.580	0.261–1.306	2.44
N-glycan					
N03	0.094–9.71	1.229	0.555	0.323–1.223	3.86
N05	0.128–9.67	1.257	0.575	0.397–1.098	3.89
N07	0.076–3.55	0.77	0.828	0.434–1.303	1.35
N12	0.054–18.8	1.929	0.502	0.225–1.010	6.16
N17	0.188–8.01	1.022	0.715	0.427–1.126	3.77
N22	0.071–6.30	1.095	0.572	0.298–1.382	2.41
N28	0.064–9.63	1.002	0.798	0.499–1.194	5.05
N31	0.022–7.86	1.168	0.587	0.295–1.179	2.57
N33	0.011–21.7	2.523	0.073	0.039–0.758	4.75
N36	0.098–7.37	0.987	0.704	0.485–1.085	3.53
N37	0.085–5.25	0.823	0.740	0.492–1.235	2.29
N42	0.119–5.34	0.806	0.773	0.529–1.190	2.58
N51	0.117–6.82	0.955	0.706	0.490–1.072	3.03
N57	0.091–6.70	1.034	0.699	0.368–1.145	2.70
N58	0.024–10.4	1.42	0.556	0.257–1.117	3.69
N66	0.024–8.37	1.258	0.556	0.278–1.193	2.94
HMO					
LNDFH-I	0.000–4.46	0.815	0.990	0.110–1.496	0.72
LNDFH-II	0.070–7.48	0.99	0.713	0.469–1.094	3.47

LNFP-I	0.002–4.48	0.952	0.768	0.258–1.454	1.32
LNFP-II	0.000–7.88	1.136	0.636	0.329–1.229	2.62
LDFT	0.000–4.59	0.822	0.956	0.393–1.352	1.33
LNT	0.028–3.57	0.643	0.901	0.557–1.359	1.02
LNnT	0.063–4.80	0.814	0.788	0.408–1.357	1.82
2'-FL	0.000–3.12	0.643	1.069	0.633–1.447	-0.03
3-FL	0.061–3.74	0.712	0.832	0.486–1.312	1.38
3'-SL	0.215–3.06	0.429	0.948	0.683–1.203	1.22
6'-SL	0.087–3.33	0.625	0.854	0.578–1.243	1.51

*The relative concentration of each glycan was normalized to a mean of 1.

** IQR, interquartile range

Table S3. Comparison of the relative concentration of N/O-glycans and HMOs between secretor milk and nonsecretor milk*

	Secretor (n=161)		Nonesecretor (n=39)		*p* value***
	median	IQR**	median	IQR	
Group A					
N07	0.823	0.434–1.359	0.856	0.439–1.108	0.691
N17	0.719	0.436–1.168	0.675	0.333–1.063	0.220
N36	0.712	0.514–1.074	0.617	0.452–1.088	0.484
N37	0.698	0.465–1.171	0.999	0.678–1.699	0.002
N42	0.828	0.540–1.257	0.619	0.419–0.853	0.009
N51	0.716	0.470–1.069	0.667	0.535–1.045	0.841
N57	0.673	0.363–1.096	0.794	0.384–1.178	0.917
N58	0.732	0.371–1.259	0.178	0.084–0.381	< 0.001
N66	0.559	0.287–1.188	0.553	0.265–1.278	0.873
N03	0.552	0.334–1.038	0.633	0.273–1.959	0.498
N05	0.571	0.397–1.056	0.609	0.404–1.201	0.496
N12	0.665	0.270–1.120	0.251	0.179–0.393	< 0.001
N22	0.627	0.299–1.388	0.501	0.274–1.201	0.349
N28	0.852	0.526–1.259	0.657	0.345–0.878	0.006
N31	0.515	0.267–0.980	1.024	0.574–1.606	0.001
N33	0.074	0.043–0.914	0.056	0.033–0.156	0.126
LNT	0.830	0.540–1.290	1.049	0.719–1.756	0.015
LNnT	0.883	0.563–1.455	0.302	0.190–0.627	< 0.001
3'-SL	0.950	0.677–1.202	0.913	0.694–1.219	0.948
6'-SL	0.821	0.574–1.210	0.919	0.613–1.299	0.423
Group B					
2'-FL	1.190	0.901–1.534	0.006	0.004–0.012	< 0.001
LDFT	1.060	0.753–1.445	0.045	0.027–0.075	< 0.001
LNFP-I	0.970	0.552–1.621	0.037	0.021–0.074	< 0.001
LNDFH-I	1.144	0.834–1.592	0.048	0.015–0.099	< 0.001
O01	0.945	0.439–1.729	0.221	0.107–0.612	< 0.001
O20	0.782	0.440–1.695	0.047	0.034–0.087	< 0.001
O28	0.674	0.390–1.561	0.070	0.037–0.261	< 0.001
Group C					
3-FL	0.706	0.452–1.087	1.993	1.268–2.397	< 0.001
LNFP-II	0.582	0.320–0.895	2.251	1.344–3.126	< 0.001

LNDFH-II	0.615	0.455–0.937	1.588	0.983–2.921	< 0.001
O08	0.456	0.182–0.902	1.361	0.502–2.468	< 0.001
O09	0.719	0.537–1.105	0.863	0.681–1.859	0.014
O17	0.667	0.370–1.109	0.576	0.327–1.301	0.709
O19	0.603	0.387–1.007	0.609	0.348–1.280	0.931
O22	0.496	0.280–1.137	0.422	0.359–1.100	0.542
O24	0.525	0.314–0.915	0.940	0.585–1.979	< 0.001
O25	0.506	0.251–1.113	0.886	0.236–1.443	0.079
O26	0.601	0.384–1.012	0.837	0.572–1.785	< 0.001
O27	0.466	0.254–1.002	0.727	0.512–1.867	0.009

*The relative concentration of each glycan was normalized to a mean of 1.

**IQR, interquartile range

*** Mann-Whitney U test
